# Integrating Biomonitoring Exposure Data into the Risk Assessment Process: Phthalates [Diethyl Phthalate and Di(2-ethylhexyl) Phthalate] as a Case Study

**DOI:** 10.1289/ehp.9059

**Published:** 2006-06-12

**Authors:** Antonia M. Calafat, Richard H. McKee

**Affiliations:** 1 Division of Laboratory Sciences, National Center for Environmental Health, Centers for Disease Control and Prevention, Atlanta, Georgia, USA; 2 Toxicology Research Task Group, Phthalate Esters Panel, American Chemistry Council, Arlington, Virginia, USA

**Keywords:** biomarkers, biomonitoring, DEHP, DEP, exposure, human, phthalate, urine

## Abstract

The probability of nonoccupational exposure to phthalates is high given their use in a vast range of consumables, including personal care products (e.g., perfumes, lotions, cosmetics), paints, industrial plastics, and certain medical devices and pharmaceuticals. Phthalates are of high interest because of their potential for human exposure and because animal toxicity studies suggest that some phthalates affect male reproductive development apparently via inhibition of androgen biosynthesis. In humans, phthalates are rapidly metabolized to their monoesters, which can be further transformed to oxidative products, conjugated, and eliminated. Phthalate metabolites have been used as biomarkers of exposure. Using urinary phthalate metabolite concentrations allows accurate assessments of human exposure because these concentrations represent an integrative measure of exposure to phthalates from multiple sources and routes. However, the health significance of this exposure is unknown. To link biomarker measurements to exposure, internal dose, or health outcome, additional information (e.g., toxicokinetics, inter- and intraindividual differences) is needed. We present a case study using diethyl phthalate and di(2-ethylhexyl) phthalate as examples to illustrate scientific approaches and their limitations, identify data gaps, and outline research needs for using biomonitoring data in the context of human health risk assessment, with an emphasis on exposure and dose. Although the vast and growing literature on phthalates research could not be covered comprehensively in this article, we made every attempt to include the most relevant publications as of the end of 2005.

The general structure of phthalates, diesters of phthalic acid, manufactured by reacting phthalic anhydride with alcohols of desired carbon-chain length, is shown in Figure 1. R and R′ are ethyl groups for diethyl phthalate (DEP) and 2-ethylhexyl groups for di(2-ethylhexyl) phthalate (DEHP). DEP (CAS no. 84-66-2) is used as a plasticizer for cellulose acetate, as a solvent, and as a carrier for fragrances in cosmetics and other consumer products [Agency for Toxic Substances and Disease Registry (ATSDR) 1995; David et al. 2001]. DEHP (CAS no. 117-81-7) is used primarily as a plasticizer in flexible vinyl, which is used in consumer products, flooring and wall coverings, food contact applications, and medical devices (ATSDR 2002; David et al. 2001).

The potential for exposure is, to a certain extent, a consequence of the physical and chemical properties of each phthalate. As molecular weight increases, vapor pressure, water solubility, and dermal uptake are reduced. The major route of human exposure for most phthalates is ingestion; exposure by inhalation, through drinking water, and via dermal contact tends to be limited (Clark et al. 2003). After ingestion, phthalates are metabolized to their corresponding hydrolytic monoesters and may further metabolize to more hydrophilic oxidative products. These metabolites can be excreted unchanged or can undergo phase II biotransformation to glucuronide conjugates (ATSDR 1995, 2002). Metabolites and not the parent diesters are likely the bioactive species (Albro 1986; Awal et al. 2004; Bility et al. 2004; Ema et al. 2003; Foster et al. 2000; Gray and Beamand 1984; Gray and Gangolli 1986; Heindel and Powell 1992; Li and Kim 2003; Saillenfait et al. 2001; Stroheker et al. 2005).

Evidence of human hazard associated with exposure to phthalates is limited, and risk assessments have been based primarily on results of animal studies. Administration of some phthalates to rodents caused liver effects, including increased weights, elevated enzyme levels, histologic changes, and tumors, associated with peroxisomal proliferation, that is, specifically with peroxisome proliferator–activated receptor α agonism (Ward et al. 1998), a process related to metabolism of cholesterol and fatty acids. Due in part to species-specific metabolic differences, the relevance of these effects to humans is questionable [International Agency for Research on Cancer (IARC) 2000; Klaunig et al. 2003]. Nevertheless, liver effects have been used to establish no observed adverse effect levels (NOAELs) for risk assessment. Evidence also exists that some phthalates and their metabolites affect reproduction and development, particularly in male rats (e.g., epididymal malformations or absence of the epididymis, testicular lesions, increased incidence of hypospadias, cryptorchidism, decreased anogenital distance, delayed preputial separation, and retention of thoracic nipples) (Barlow et al. 2004; Barlow and Foster 2003; Carruthers and Foster 2005; Corton and Lapinskas 2005; Ema and Miyawaki 2001; Fisher 2004; Foster 2005; Gray et al. 2000; Mylchreest et al. 1998), apparently by a process involving inhibition of androgen biosynthesis (Parks et al. 2000).

Because DEP is used in personal care products, dermal toxicity is of interest. Primary dermal irritation with undiluted DEP has not been reported in humans (Api 2001). DEP was not a dermal sensitizer in healthy human volunteers, although sensitization was reported in some studies, mostly involving persons with skin diseases (Api 2001). No reports exist of oral or inhalation toxicity of DEP or of any adverse effects in humans exposed exclusively to DEP (ATSDR 1995). The oral reference dose (RfD) for DEP, 800 μg/kg/day, was derived from a NOAEL of 750 mg/kg/day based on reduced growth rate, food consumption, and increased organ weights in rats [U.S. Environmental Protection Agency (U.S. EPA) 1993b]. No evidence of other effects in animals at lower NOAELs exists (Api 2001; Barber et al. 2000; Gray et al. 2000).

Information on the oral toxicity of DEHP is limited to mild abdominal pain and diarrhea in two persons who ingested single large doses (ATSDR 2002). No reports exist of dermal or inhalation toxicity of DEHP in adult humans, and DEHP is neither a dermal irritant nor a sensitizer (ATSDR 2002; Medeiros et al. 1999). DEHP does not appear to be readily absorbed through human skin (ATSDR 2002). Lung disorders, resembling hyaline membrane disease, were observed in three newborns who, as preterm infants, received ventilation therapy involving polyvinyl chloride tubing (ATSDR 2002). The U.S. EPA classifies DEHP as a probable human carcinogen (B2) and, based on evidence of increased liver weight in rodents, established the RfD at 20 μg/kg/day (U.S. EPA 1993a, 2002). IARC (2000) revised its classification from “probable” to “not classifiable” after determining that the mode of action was irrelevant to humans. Because of the controversy regarding relevance of DEHP-induced rodent liver cancer to humans, cancer risk will not be discussed in this article.

In recent years the potential reproductive and developmental effects of DEHP have received more attention than the carcinogenic effects. In particular, developing rats are more sensitive to the testicular toxicity of DEHP than are older animals [Center for the Evaluation of Risks to Human Reproduction (CERHR) 2005; Kavlock et al. 2002]. Exposure of rats to DEHP during the late gestational period affected male reproductive development with a NOAEL of 5–8 mg/kg body weight/day. This NOAEL was used to assess the potential for human reproductive risks associated with DEHP exposure (CERHR 2005). Similarly, a previously determined NOAEL of 3.7 mg/kg/day for testicular effects was used by the European Union’s Scientific Committee for Toxicity, Ecotoxicity, and the Environment (CSTEE) as the basis for a tolerable daily intake (TDI) of 37 μg/kg/day (CSTEE 1998).

## Biomarkers of Exposure

Phthalates are widely used in laboratory equipment, and contamination is possible (Blount et al. 2000a; Kessler et al. 2001). Sample contamination problems are greatly minimized when phthalate metabolites are measured (Blount et al. 2000a). To select the most appropriate biomarkers of exposure, understanding the toxicokinetics of individual phthalates is fundamental. Although differences in absorption of phthalates exist, we address only metabolic differences in this article.

In rats, monoethyl phthalate (MEP) is the principal urinary metabolite of DEP; smaller amounts of phthalic acid and DEP are also found (Albro and Moore 1974). Metabolism in humans is assumed to be similar (ATSDR 1995). Elimination half-lives of DEP and MEP have not been experimentally defined but, like DEHP and its hydrolytic metabolite mono(2-ethylhexyl) phthalate (MEHP), are assumed to be a few hours. These findings suggest that MEP is the most sensitive and specific biomarker of exposure to DEP.

More than 20 urinary metabolites of DEHP have been proposed (Albro 1986). In rodents these consist primarily of terminal oxidation products. In humans the principal DEHP metabolites are side-chain–oxidized metabolites of MEHP (Koch et al. 2004a, 2005b). In two cancer patients receiving an infusion of a platelet concentrate containing DEHP, > 50% of the DEHP disappeared from the blood in about 30 min and appeared as DEHP derivatives in urine within 6 hr (Peck and Albro 1982). In another study of two volunteers who received DEHP orally, the urinary elimination half-life of DEHP was estimated to be 12 hr (Schmid and Schlatter 1985). The urinary excretion of DEHP metabolites in one person after three oral doses of D_4_-DEHP followed a multiphase elimination model (Koch et al. 2004a, 2005b). For the first 4–8 hr, excretion half-lives were approximately 2 hr for mono(2-ethyl-5-hydroxyhexyl) phthalate (MEHHP), mono(2-ethyl-5-oxohexyl) phthalate (MEOHP), and MEHP. Fourteen to eighteen hours postadministration, half-lives were 5 hr (MEHP) and 10 hr (MEHHP and MEOHP) (Koch et al. 2004a). MEHHP was the major metabolite initially; other metabolites, mono(2-ethyl-5-carboxypentyl) phthalate and mono(2-carboxymethylpentyl) phthalate, were more abundant starting 12 hr after exposure (Koch et al. 2005b). The higher urinary concentrations in humans of MEOHP and MEHHP than of MEHP (Barr et al. 2003; Kato et al. 2004; Koch et al. 2003c, 2004b, 2005b; Silva et al. 2006a, 2006b) suggest that oxidative metabolites may provide greater analytical sensitivity than MEHP. Furthermore, oxidative metabolites cannot be formed as a result of sampling contamination and may be more advantageous as biomarkers of exposure to DEHP than MEHP. DEHP, seldom found in blood or urine except as a consequence of contamination, is not recommended as a biomarker in studies involving these media but may be useful in studies involving other media (e.g., feces).

Highly specific, sensitive, accurate, and precise analytical methods using isotope-dilution–high-performance liquid chromatography (HPLC) coupled with tandem mass spectrometry for measuring parts-per-billion levels of selected phthalate metabolites in biologic matrices have been described (Blount et al. 2000a; Calafat et al. 2004b; Kato et al. 2003a, 2003b, 2003c, 2005; Koch et al. 2003b, 2004a; Mortensen et al. 2005; Preuss et al. 2005; Silva et al. 2003, 2004c, 2005a, 2005b; Takatori et al. 2004).

Urine (as matrix) and phthalate metabolite concentrations (as biomarkers) represent the most common approach to investigating phthalate exposure in humans. Phthalate concentrations in blood have been reported, but most assessed concentrations of diesters. Data from such studies are often questionable because of the potential for diester contamination. Consequently, methods were developed to measure concentrations of metabolites in serum (Kato et al. 2003bKato et al. 2004a; Silva et al. 2005b; Takatori et al. 2004), breast milk (Calafat et al. 2004b; Mortensen et al. 2005), saliva (Silva et al. 2005a), and human amniotic fluid (Silva et al. 2004b). Data from media other than urine could also be used for exposure assessment, but it might be more difficult to collect the samples. Thus, these alternative media may not readily lend themselves to large screening programs but may be useful in specific situations.

## Environmental Public Health Uses of Biomonitoring Data

Defining human exposure to phthalates requires measuring concentrations of parent compounds or their metabolites in urine and other biomatrices as well as understanding the pharmacokinetics of individual phthalates. The Centers for Disease Control and Prevention (CDC) collects urinary metabolite data for the general population, primarily through the National Health and Nutrition Examination Survey (NHANES), an ongoing national survey designed to evaluate the health and nutritional status of the U.S. population. NHANES is unique in its ability to examine public health issues that can be addressed through physical and laboratory examinations. NHANES 1999–2000 and 2001–2002 (CDC 2005; Silva et al. 2004a) provided nationally representative population-based urinary phthalate metabolite data, based on one specimen per participant, for selected demographic groups in the United States. However, young (i.e., < 6 years of age) and older individuals (i.e., > 60 years of age) were not represented in the population sampled, and no data on prenatal exposures were collected.

Data from NHANES and other studies conducted in the United States (Adibi et al. 2003; Blount et al. 2000b; Brock et al. 2002; CDC 2005; Hoppin et al. 2002; Silva et al. 2004a) and abroad (Koch et al. 2003c, 2004b) have confirmed that human exposure to phthalates is widespread (Tables 1–3). Some situations, not specifically addressed by large surveys such as NHANES, may lead to phthalate exposures well above those found in the general population. Examples include the use of certain medications with enteric coatings containing phthalates [e.g., DEP, dibutyl phthalate (DBP)] (Hauser et al. 2004a; Koch et al. 2005d) or related to using DEHP in medical devices (Calafat et al. 2004a; Green et al. 2005; Koch et al. 2005a, 2005c).

Studies of specific health effects with environmental phthalate exposures using urinary metabolite concentrations as exposure surrogates exist (Duty et al. 2003a, 2003b, 2004, 2005; Hoppin et al. 2004; Jonsson et al. 2005; Swan et al. 2005). However, these epidemiologic data are limited and drawing firm conclusions has been difficult (Hauser and Calafat 2005).

## Internal Dose and Exposure Assessment

Previous exposure assessments for phthalates have been indirect, that is, relying on surveys of product use, measuring phthalates in various media, estimating human contact, and pharmacokinetic assumptions based on animal data. In contrast, direct methods using urinary metabolite concentrations as biomarkers for phthalate exposure may provide the most accurate assessments because these concentrations represent an integrative measure of exposure from multiple sources and routes and can be used to calculate phthalate exposure in the general (Blount et al. 2000b; CDC 2005; Koch et al. 2003c; Silva et al. 2004a) and specific populations (Adibi et al. 2003; Brock et al. 2002; Duty et al. 2003a, 2003b, 2004; Hoppin et al. 2002; Jonsson et al. 2005; Koch et al. 2004b; Swan et al. 2005).

For phthalate metabolite data, two calculation methods produced similar results (David 2000; Kohn et al. 2000). For illustrative purposes, we show the method of David (2000) as expressed by Koch et al. (2003a):


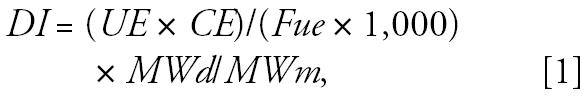


in which *DI* is the daily intake in milligrams per kilogram per day; *UE* is the creatinine-corrected urinary metabolite concentration in micrograms per gram; *CE* is the creatinine clearance rate, normalized for body weight, in milligrams per kilogram per day; *Fue* is the molar conversion factor that relates urinary excretion of metabolite to diester ingested; and *MWd* and *MWm* are the molecular weights of diester and metabolite, respectively. For these calculations, we set CE at 20 mg/kg/day for adults, 11 mg/kg/day for children, and 9.8 mg/kg/day for infants (Jacobs et al. 2001; Tietz 1990). We set Fue at 0.69 mg/kg/day for DEP (as MEP), 0.13 mg/kg/day for DEHP (as MEHP), 0.23 mg/kg/day (as MEHHP), and 0.15 mg/kg/day (as MEOHP).

An Fue value for DEP has not been determined experimentally but is assumed to be similar to the value determined for DBP (Anderson et al. 2001). By contrast, urinary excretion of DEHP metabolites has been studied after oral (Anderson et al. 2001; Koch et al. 2004a; Schmid and Schlatter 1985) and intravenous (Peck and Albro 1982) administration. The earliest reports of Fue for DEHP metabolites came from studies that had either analytical limitations or small sample sizes (Peck and Albro 1982; Schmid and Schlatter 1985). Subsequently, an MEHP Fue value was determined by HPLC–mass spectrometry from a study involving seven individuals dosed orally with both ^13^C-DEHP and ^13^C-diisooctyl phthalate (Anderson et al. 2001). Because the ^13^C-MEHP and ^13^C-monooctyl phthalate signals co-eluted, Fue for these species could not be determined separately, and the MEHP value of 0.13 is the average (Anderson et al. 2001). We used Fue for the oxidative DEHP metabolites (MEHHP, 0.23; MEOHP, 0.15) from a study of one adult man given three single oral doses of D_4_-DEHP; the estimated Fue for MEHP was 0.06 (Koch et al. 2005b), about half the value used in the calculations in this case study.

The first data on urinary phthalate metabolite concentrations, including MEP and MEHP, reported in a U.S. population of 289 adults from NHANES III (Blount et al. 2000b), were used to calculate exposures to the corresponding phthalate diesters (David 2000; Kohn et al. 2000). Subsequently, the CDC reported U.S. nationally representative urinary concentrations of seven phthalate metabolites in 2,540 participants of NHANES 1999–2000 (Silva et al. 2004a) and of 10 phthalate metabolites in 2,782 participants of NHANES 2001–2002 (CDC 2005). The frequencies of detection of individual phthalate metabolites were similar. However, the median concentration of MEP was almost 2-fold lower in NHANES 1999–2000 and 2001–2002 than in NHANES III. These differences may have reflected reduced exposures to DEP or have been related to differences in sample sizes. In contrast, the MEHP concentrations remained essentially constant, although they were highest in NHANES 2001–2002 (Table 1). MEHHP and MEOHP were only measured in NHANES 2001–2002. Their median concentrations were 5-fold (MEHHP) and more than 3-fold (MEOHP) higher than the median MEHP concentration. The NHANES 1999–2000 and 2001–2002 data, stratified by age, gender, or ethnicity, indicated some differences in urinary concentrations of phthalate metabolites (CDC 2005; Silva et al. 2004a). For MEHP, MEHHP, and MEOHP, children exhibited higher urinary concentrations than adults, although when accounting for creatinine clearance, the calculated external exposures were similar (Table 2).

Urinary concentrations of DEP and DEHP metabolites in other smaller groups (Adibi et al. 2003; Brock et al. 2002; Duty et al. 2004; Hoppin et al. 2002; Koch et al. 2003c, 2004b) were largely consistent with the NHANES 1999–2002 data (Table 3). In general, differences between various segments of the population were smaller than the differences across the population, that is, from lowest to the most highly exposed individuals. The underlying explanation for the range of exposures is unknown but may be related to individual lifestyle choices. However, selection of study subjects (at least for NHANES) did not exclude those occupationally exposed, and specific situations may contribute to higher exposures for some individuals (Calafat et al. 2004a; Green et al. 2005; Hauser et al. 2004a; Koch et al. 2005a, 2005d, 2005c). Median urinary MEP concentrations in 85 German children and adults were approximately half those in NHANES 1999–2002 (Koch et al. 2003c, 2004b). By contrast, median urinary MEHP, MEHHP, and MEOHP concentrations were approximately twice those in NHANES 1999–2002, but 95th percentile values were similar (Becker et al. 2004; Koch et al. 2003c, 2004b) (Table 1). Whether these findings reflect differences in sampling (e.g., first morning vs. non-first morning voids, nonrepresentative nature of the population examined in Germany) or in exposure patterns between the United States and Germany is unknown.

Estimates of DEP exposure resulting from its use in personal care products, based on conservative assumptions, were not realistic (730 μg/kg/day from fragrances and 100 μg/kg/day from personal care products) (Api 2001). With food as the largest identified contributor to exposure for most individuals, calculated median DEP exposure ranges were 2–6 μg/kg/day for most of the population, with somewhat higher estimates for toddlers and lower estimates for infants (Clark et al. 2003). For DEHP, relying heavily on a previous study (Huber et al. 1996), estimated DEHP exposure ranges within the general population were 3–30 μg/kg/day, with higher exposures likely in occupational settings and the highest associated with certain medical procedures (Doull et al. 1999). Estimates from other researchers (Clark et al. 2003; Meek and Chan 1994) also fall in this range.

For DEP a comparison of the biomarker-based and indirect approaches indicates that, in adults, mean estimates derived by indirect methods (Clark et al. 2003) were about half the mean exposures calculated from bio-marker-based data (Table 4). Because this indirect approach did not consider DEP exposure from cosmetics use, these differences are expected. The 95th percentile exposures calculated from biomonitoring data were above these indirect estimates but far below unrealistic estimates of exposures from cosmetic and personal care products (Api 2001). One might hypothesize that exposure from sources other than personal care products accounts for approximately half the mean total DEP exposure, with exposure from personal care products comprising the remainder. In agreement with this hypothesis, children have lower exposures to DEP than adults (Tables 1, 2). Three overall conclusions emerge from this example: *a*) indirect methods can provide realistic estimates of exposure only if reasonable assumptions are used; *b*) use of biomonitoring data can yield precise exposure estimates because it does not require overly conservative assumptions; and *c*) it may identify situations in which not all potential sources of exposure were considered.

For DEHP, urinary MEHP data produced estimates of mean exposure that were lower than those using the indirect methods, although the 95th percentile values were similar (Table 4). Using DEHP oxidative metabolite data, the estimated DEHP exposures are about twice those calculated from MEHP data (Tables 1, 2, 4). That mean DEHP exposures within the general population, calculated from urinary metabolite data, are approximately 4-fold lower than the indirect estimates may be due, in part, to reliance on older measurements of phthalates in various media, particularly food, as the basis for indirect estimates (Clark et al. 2003). Conservatism may also be introduced by assumptions about absorption based on results of animal studies. Nevertheless, this comparison suggests that, for DEHP, all relevant sources of exposure were taken into consideration when using the indirect approach.

## Risk Assessment

Biomonitoring data can also be used to address the exposure component of risk assessment. In risk assessment, exposure estimates are compared with NOAELs that for phthalates were from studies in rats. Important and controversial issues relating to these hazard data include choice of species, identification of critical end points, and relevance to humans (Bosgra et al. 2005; Foster 2005). Discussing those issues in detail is beyond the scope of this article. Rather, this section relates results of risk assessments based on phthalate exposures calculated from urinary metabolite data to conclusions of previous risk assessments.

The most reasonable indirect estimates of mean exposure to DEP were 2–6 μg/kg/day, depending on the ages of the groups considered and neglecting consideration of cosmetics and personal care products (Clark et al. 2003). Estimates of DEP exposure in the general population, based on biomonitoring data, are 5.4 μg/kg/day, with a 95th percentile of 64.7 μg/kg/day (Table 4). Thus, indirect and biomarker-based methods produced comparable estimates and indicated that within the United States most individuals are exposed to DEP levels well below the RfD (800 μg/kg/day).

For DEHP, indirect estimates of mean exposure were 5.8–8.2 μg/kg/day (Clark et al. 2003) and a range of 3–30 μg/kg/day (Doull et al. 1999). From urinary metabolite data, estimated mean exposures are in the range of 1–2 μg/kg/day, with a 95th percentile of 7–17 μg/kg/day depending on the metabolite used (Table 4). This comparison suggests that both indirect and biomarker-based methods produced mean estimates below the RfD (20 μg/kg/day) and TDI (37 μg/kg/day), although the upper ranges of exposure approximated the RfD. As another example, the National Toxicology Program (NTP) CERHR determined that the NOAEL for reproductive effects in rats was 5–8 mg/kg/day (CERHR 2005) and expressed concern over the potential for reproductive risk among infants younger than 1 year, if their exposures were significantly higher than those of the general population (1–30 μg/kg/day). Biomonitoring data are unavailable for healthy infants younger than 1 year, so this specific question cannot be addressed from the available data. However, for those 6 or more years of age, biomonitoring data indicate that ambient exposures to DEHP within the United States are lower than estimates used by the NTP-CERHR and that children’s and adults’ exposures are comparable. Some medical interventions may result in higher exposures to DEHP (Calafat et al. 2004a; Green et al. 2005; Koch et al. 2005a, 2005c). These medical treatments entail risk–benefit calculations that make risk assessments substantially different from those relating to ambient exposures (U.S. Food and Drug Administration 2001) and are beyond the scope of this exercise. Note that for children and adults, exposure estimates calculated from the oxidized DEHP metabolites were approximately twice those calculated from MEHP (Tables 1, 2). However, for premature neonates the differences were in the range of an order of magnitude (Table 2), presumably from differences in metabolism and/or excretion in these preterm infants.

## Recommendations for Future Research

We make the following recommendations for future research:

- *Improve the understanding of human metabolism and pharmacokinetics.* The most relevant urinary metabolites and appropriate metabolites for other matrices that provide the greatest analytical sensitivity must be measured. Differences in metabolic patterns among phthalates are important both toxicologically and in exposure assessment, especially when comparing relative exposures to different phthalates because the complex metabolism of high-molecular-weight phthalates leads to additional metabolic products (e.g., oxidative metabolites).
- *Refine molar conversion factors to relate external phthalate exposure to urinary metabolite concentrations.* Based on available data, the largest uncertainties appear in premature neonates.
- *Determine the biologic media best suited for biomarker studies.* If media other than urine are evaluated, methodologic issues must be considered.
- Improve the understanding of the mechanisms of action of phthalates in humans.
- *Determine whether more highly exposed groups can be identified and, if so, identify the sources of exposure.* Potentially vulnerable segments of the population (e.g., children, women of reproductive age, minorities) should be evaluated.
- *Determine whether use of urinary metabolite data as an adjunct to epidemiology studies is possible.* One specific issue relates to categorizing exposure from a limited number of urine samples (Hauser et al. 2004b; Hoppin et al. 2002).

## Figures and Tables

**Figure 1:**
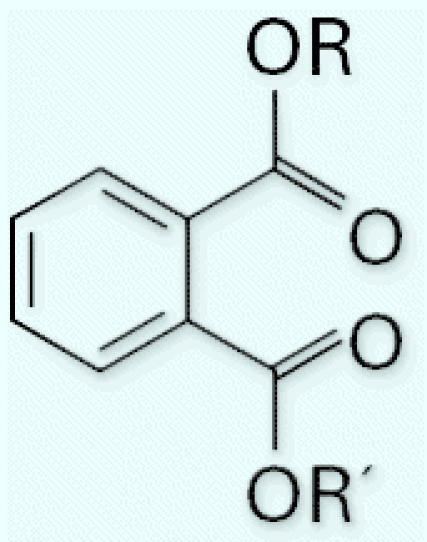
Generic chemical structure of phthalates. R and R′ are ethyl groups for DEP and 2-ethylhexyl groups for DEHP.

**Table 1:** Urinary concentrations (micrograms per gram creatinine) of MEP, MEHP, MEHHP, and MEOHP and estimated exposures (in parentheses, micrograms per kilogram per day) to DEP and DEHP calculated using urinary concentrations from several studies of adults or the general population.

	Geometric mean				95th percentile			
	DEP		DEHP		DEP		DEHP	
Population group	MEP	MEHP	MEHHP	MEOHP	MEP	MEHP	MEHHP	MEOHP
289 adults (Blount et al. 2000b)	345 (11.4)	3.0 (0.5)	ND	ND	2,610 (86.6)	15.2 (3.3)	ND	ND
2,536 persons 6 to > 20 years of age (Silva et al. 2004a)	163 (5.4)	3.12 (0.7)	ND	ND	1,950 (64.7)	18.5 (4.0)	ND	ND
2,772 persons 6 to > 20 years of age (CDC 2005)	167 (5.5)	3.99 (0.9)	18.8 (2.1)	12.6 (2.2)	1,860 (61.7)	32.8 (7.1)	147 (16.8)	87.5 (15.6)
85 children and adults (Koch et al. 2003c)^a^	165^b^ (5.5)	12.4^b^ (2.7)	57.2^b^ (6.5)	41.7^b^ (7.4)	673 (22.2)	34.7 (7.5)	143 (16.3)	106 (18.9)

ND, not determined.

a In their calculations of exposure, Koch et al. (2003a, 2003c) used different Fue and CE values. We recalculated the estimated exposures using the factors listed in the text, for comparison with other studies included in this table.

b Mean value.

**Table 2:** Urinary concentrations (micrograms per gram creatinine) of MEP, MEHP, MEHHP, and MEOHP and estimated exposures (in parentheses, micrograms per kilogram per day) to DEP and DEHP calculated using urinary concentrations from several studies of children.

	Geometric mean				95th percentile			
	DEP		DEHP		DEP		DEHP	
Population group	MEP	MEHP	MEHHP	MEOHP	MEP	MEHP	MEHHP	MEOHP
328 children 6–11 years of age (Silva et al. 2004a)	92.6 (1.7)	5.19 (0.6)	ND	ND	625 (11.4)	41.9 (5.0)	ND	ND
392 children 6–11 years of age (CDC 2005)	96.9 (1.8)	5.02 (0.6)	38.3 (2.4)	26.6 (2.6)	837 (15.3)	31.2 (3.7)	211 (13.2)	130 (12.8)
254 children 3–14 years of age (Becker et al. 2004)	ND	6.2 (0.7)	40.7 (2.6)	31.2 (3.1)	ND	23.7 (2.8)	170 (10.7)	119 (11.7)
36 children < 7 years of age (Koch et al. 2004b)^a^	ND	8.7^b^ (1.0)	55.8^b^ (3.5)	38.3^b^ (3.8)	ND	27.5 (3.3)	113 (7.1)	75.8 (7.4)
19 children 12–18 months of age (Brock et al. 2002)^c^	184.1^b^ (6.3)	4.6^b^ (2.8)	ND	ND	ND	ND	ND	ND
6 premature neonates (Calafat et al. 2004a)	ND	800 (85.0)	16,634 (931)	14,351 (1,256)	ND	6,043 (641)	62,982 (3,523)	52,189 (4,566)

ND, not determined.

a In their calculations of exposure, Koch et al. (2004b) used different Fue and CE values. We recalculated the estimated exposures using the factors listed in the text, for comparison with other studies included in this table.

b Mean value.

c Urinary concentrations are in nanograms per milliliter (Brock et al. 2002). Estimated doses are from Clark et al. (2003) using the published individual values for urinary creatinine (milligrams per deciliter) (Brock et al. 2002), and molar conversion factors of 0.64 (MEP) and 0.14 (MEHP).

**Table 3:** Urinary concentrations (micrograms per gram creatinine) of MEP, MEHP, MEHHP, and MEOHP and estimated exposures (in parentheses, micrograms per kilogram per day) to DEP and DEHP calculated using urinary concentrations from specific populations.

	Geometric mean				95th percentile			
	DEP		DEHP		DEP		DEHP	
Population group	MEP	MEHP	MEHHP	MEOHP	MEP	MEHP	MEHHP	MEOHP
35 African-American women (Hoppin et al. 2002)	183^a^ (6.0)	12.3^a^ (2.7)	ND	ND	611^b^ (20.2)	77.3^b^ (16.7)	ND	ND
702 non-Hispanic blacks (CDC 2005)	247 (8.2)	4.63 (1.0)	21.0 (2.4)	13.8 (2.5)	2,070 (68.7)	39.8 (8.6)	161 (18.4)	101 (18.0)
1,405 females 6–60 years of age (CDC 2005)	187 (6.2)	4.53 (1.0)	19.7 (2.2)	13.5 (2.4)	1,430 (47.4)	35.1 (7.6)	160 (18.3)	92.3 (16.5)
25 pregnant women (Adibi et al. 2003)	690^a^ (22.9)	40.5^a^ (8.8)	ND	ND	5,520^b^ (183.1)	449^b^ (97.4)	ND	ND
220 men (Duty et al. 2004)^c^	183.1 (6.1)	7.0 (1.5)	ND	ND	2,002.1 (66.4)	130.9 (28.4)	ND	ND
1,367 males 6–60 years of age (CDC 2005)	147 (4.9)	3.49 (0.8)	17.9 (2.0)	11.8 (2.1)	2,080 (69.0)	31.2 (6.8)	136 (15.5)	83.1 (14.8)
19 adults (Koch et al. 2004b)^d^	ND	8.6^e^ (1.9)	28.1^e^ (3.2)	17.2^e^ (3.1)	ND	24.7 (5.4)	48 (5.5)	34.7 (6.2)

ND, not determined.

a Mean value.

b Maximum value.

c Urinary concentrations were corrected using specific gravity instead of creatinine.

d In their calculations of exposure, Koch et al. (2004b) used different Fue and CE values. We recalculated the estimated exposures using the factors listed in the text, for comparison with other studies included in this table.

e Median value.

**Table 4:** Estimates of the geometric mean (95th percentiles in parentheses) exposures (in micrograms per kilogram per day) to DEP and DEHP using the geometric mean (95th percentile) urinary phthalate metabolite concentrations compared with indirect estimates based on phthalate diester levels in various media (e.g., food, air, water, soil, and dust).^a^

	DEP		DEHP	
Population group	Biomarker data	Indirect estimate	Biomarker data	Indirect estimate
2,772 persons 6 to > 20 years of age (CDC 2005)	5.5 (61.7)	2.5 730^c^	0.9 (7.1)^b^ 2.1 (16.8)^d^ 2.2 (15.6)^e^	8.2
742 adolescents 12–19 years of age (CDC 2005)	5.0 (44.1)	3.0	0.8 (5.5)^b^ 2.2 (11.6)^d^ 2.4 (12.6)^e^	10.0
392 children 6–11 years of age (CDC 2005)	1.8 (15.3)	5.7	0.6 (3.7)^b^ 2.4 (13.2)^d^ 2.6 (12.8)^e^	18.9
254 children 3–14 years of age (Becker et al. 2004)	ND		0.7 (2.8)^b^ 2.6 (10.7)^d^ 3.1 (11.7)^e^	
19 children 12–18 months of age (Brock et al. 2002)^f^	6.3^g^	10.6	2.8^g^	25.8

ND, not determined.

a Data from Clark et al. (2003).

b Using MEHP data.

c Data from Api (2001).

d Using MEHHP data.

e Using MEOHP data.

f The age of the children for the indirect estimate calculations was 7 months to 4 years.

g Estimated doses are from Clark et al. (2003) using the published individual values for urinary creatinine (milligrams per deciliter) and mean urinary phthalate metabolite concentrations (nanograms per milliliter) (Brock et al. 2002) and molar conversion factors of 0.64 (MEP) and 0.14 (MEHP).
